# Collaborative multicenter logistics delivery network optimization with resource sharing

**DOI:** 10.1371/journal.pone.0242555

**Published:** 2020-11-23

**Authors:** Shejun Deng, Yingying Yuan, Yong Wang, Haizhong Wang, Charles Koll

**Affiliations:** 1 College of Civil Science and Engineering, Yangzhou University, Yangzhou, China; 2 School of Management, Shanghai University, Shanghai, China; 3 School of Economics and Management, Chongqing Jiaotong University, Chongqing, China; 4 School of Civil and Construction Engineering, Oregon State University, Corvallis, OR, United States of America; Chang Gung University, TAIWAN

## Abstract

Collaboration among logistics facilities in a multicenter logistics delivery network can significantly improve the utilization of logistics resources through resource sharing including logistics facilities, vehicles, and customer services. This study proposes and tests different resource sharing schemes to solve the optimization problem of a collaborative multicenter logistics delivery network based on resource sharing (CMCLDN-RS). The CMCLDN-RS problem aims to establish a collaborative mechanism of allocating logistics resources in a manner that improves the operational efficiency of a logistics network. A bi-objective optimization model is proposed with consideration of various resource sharing schemes in multiple service periods to minimize the total cost and number of vehicles. An adaptive grid particle swarm optimization (AGPSO) algorithm based on customer clustering is devised to solve the CMCLDN-RS problem and find Pareto optimal solutions. An effective elite iteration and selective endowment mechanism is designed for the algorithm to combine global and local search to improve search capabilities. The solution of CMCLDN-RS guarantees that cost savings are fairly allocated to the collaborative participants through a suitable profit allocation model. Compared with the computation performance of the existing nondominated sorting genetic algorithm-II and multi-objective evolutionary algorithm, AGPSO is more computationally efficient. An empirical case study in Chengdu, China suggests that the proposed collaborative mechanism with resource sharing can effectively reduce total operational costs and number of vehicles, thereby enhancing the operational efficiency of the logistics network.

## Introduction

With the development of e-commerce and continuous improvement of global living standards, the surge in the demand for consumer product delivery has led to increasingly complex logistics networks. Such demand is difficult to accommodate with existing logistics resources, especially during major festivals and events. In 2009 when Alibaba launched the Double Eleven Shopping Festival, the number of parcels delivered was approximately 260,000; in 2019, the number was 1.292 billion, a 5000-fold increase [1]. In response to this situation, logistics enterprises should adopt effective resource sharing and collaborative mechanisms to reduce the occurrence of delayed delivery, damage, or missing packages during the delivery process.

The traditional multi-depot vehicle routing problem (MDVRP) was proposed to address the situation where a large number of customers are served by multiple depots through a series of vehicles, which aims to optimize the complex structure of logistics networks [2, 3]. Unfortunately, with increasing customer demands, combined with rising transportation costs and limited logistics resources, the disadvantages of independent operation of distribution centers (DCs) are impossible to overlook. Collaboration among DCs enables vehicles to achieve reasonable resource sharing among multiple DCs and service periods [4]. The study of CMDVRP aims to identify and validate a collaborative mechanism among DCs to reduce the total cost of multicenter logistics distribution through collaboration, and thus improve the overall operational efficiency of the logistics network. The optimization problem of collaborative multicenter logistics delivery networks based on resource sharing (CMCLDN-RS) is a logical extension of CMDVRP. It considers resource sharing within and across service periods and can seek to find a collaborative mechanism involving different resource sharing schemes for logistics networks to reduce total operational costs, which effectively improves the reliability and stability of the logistics network.

In this study, a collaborative mechanism is designed to promote the efficiency of a multicenter logistics delivery network. The mechanism considers various resource sharing schemes to reduce total operational costs. A bi-objective integer programming model based on the most suitable resource sharing schemes is developed to minimize the total logistics operational costs and number of vehicles. An adaptive grid particle swarm optimization (AGPSO) algorithm integrated with customer clustering is uniquely created to address the CMCLDN-RS problem. The elite iteration process is incorporated into the improved hybrid algorithm, which enhances the local and global search capabilities of the algorithm. By comparing different profit allocation schemes, the proposed profit allocation strategy and orders that DCs join a collaborative alliance are considered during the process of finding a stable collaborative alliance based on vehicle sharing among different service periods.

The remaining sections of this paper are organized as follows. In Section 2, related literature is reviewed. In Section 3, the problem of CMCLDN-RS is stated and explained in detail. In Section 4, a bi-objective mixed-integer linear programing model based on resource sharing is established for CMCLDN-RS to minimize total logistics operational costs. In Section 5, an improved MOPSO algorithm is proposed to obtain the optimal route for serving customers. In Section 6, a case study is conducted to test the applicability to CMCLDN-RS. In Section 7, remarks and directions for future research are suggested.

## Literature review

The collaborative multicenter delivery logistics network optimization with resource sharing is a further discussion of the research for traditional MDVRP with time windows (MDVRPTW) and collaborative logistics network optimization. It considers the synergy among participants with the basis of MDVRPTW. Related research for collaborative MDVRPTW is illustrated in Subsection 2.1, MDVRP optimization with resource sharing is presented in Subsection 2.2, relevant solution methods and objectives for CMCVRP-RS are shown in Subsection 2.3, and profit allocation in collaborative logistics networks is proposed in Subsection 2.4.

### Collaborative MDVRPTW

Traditional MDVRPTW aims to find optimal routes for serving a set of customers with different requests under time windows among multiple depots [5–7]. The collaboration between different depots is considered to achieve resource sharing in collaborative MDVRPTW. Nadarajah and Bookbinder [8] suggested integrating trucks into the collaborative depots to avoid situations in which the trucks were traveling with less than full loads. Wang et al. [9] optimized a collaborative distribution network to obtain oil distribution routes efficiently, which minimized the total operating cost to solve the half open MDVRP. Li et al. [10] proposed that depots could form alliances to exchange transportation requests in a collaborative logistics network, which maximizes the total profit in MDVRPTW. Vaziri et al. [11] focused on the collaboration between multiple depots serving customers with different commodity requirements and developed a mixed-integer programming model to solve MDVRPTW. Previous studies on collaborative MDVRPTW have demonstrated the benefits of cooperation, but collaborative approaches can be diverse.

### MDVRP optimization with resource sharing

MDVRP optimization is an important component of the entire logistics network optimization process, and transportation resource sharing has been considered in many studies [12]. Lin [13] coordinated transportation resources to improve the efficiency of the pickup and delivery logistics network, and was able to increase the benefits and usage of vehicles. Liu et al. [2] considered vehicle sharing in a collaborative network to tackle a multi-depot capacitated vehicle routing problem. Wen and Sun [14] proposed to optimize collaborative transport by sharing fleets. Fernandez et al. [15] promoted the collaboration among depots to serve shared customers, which reduced the overall logistics operational cost. Cortes and Suzuki [16] considered transportation resource sharing of certain customers in the delivery process and obtained 10% cost savings due to the consolidation of different vehicles.

In a collaborative logistics network, constructing proper models for optimizing the network with a basis of resource sharing is essential [17, 18]. Lyu et al. [19] established a mathematical model which considered vehicle capacity sharing to optimize the transportation operations in a collaborative logistics network. Neves-Moreira et al. [20] developed a novel mathematical model for the freight transportation problem, which aimed to ensure resource synchronization among multiple nodes. Guajardo et al. [21] proposed a mixed-integer linear programming model to minimize the total transportation costs in solving the collaborative alliance configuration problem. Chen et al. [22] considered logistics resource sharing and presented a collaborative model based on the vehicle routing problem. Although collaboration among centers has been considered in many studies, knowledge gaps still remain in simultaneous sharing of multiple resources.

### Related solution methods and objectives for CMCVRP-RS

The research to solve the corresponding problems provides a reference for studying MCVRP in a collaborative network based on resource sharing [18, 23]. Relevant solution methods and objectives for CMCVRP-RS are shown in Table 1. The acronyms of relevant research are defined as follows:

PDPTW: Pickup and delivery problem with time windows
GLNC: Global logistics network configurations
CLN: Collaborative logistics network
CSCL: Coalition structure in collaborative logistics
CLNO: Collaborative logistics network optimization
MDVRP: Multi-depot vehicle routing problem
MDVRPTW: Multi-depot vehicle routing problem with time windows
LCT: Logistics collaboration considering trust
CCDP: Carrier collaboration decision-making problem
MDVTRSP: Multi-depot vehicle type rescheduling problem
CVRP: Collaborative vehicle routing problem with resource sharing

**Table 1:** Comparison of relevant solution methods and objective functions for CMCVRP-RS.

Reference in chronological order	Acronym of problem studied	Objective function	Solution method
Ropke and Pisinger [24]	PDPTW	Construct routes visiting all locations	Adaptive Large Neighborhood Search Heuristic
Sheu and Lin [25]	GLNC	Minimize network configuration costs	Statistics and analysis
Hafezalkotob and Makui [26]	CLN	Maximize flow problem	Game theory
Guajardo and Rönnqvist [12]	CSCL	Minimize total cost among participants	Design coalition structure
Xu et al. [27]	CLNO	Obtain high stability and low cost	Expected value model and orthogonal experiment design method
Li et al. [10]	MDVRPTW	Minimize total traveling costs	Hybrid genetic algorithm
Bae and Moon [28]	MDVRPTW	Minimize fixed costs of depots and delivery expenses	Heuristic and genetic algorithms
Daudi et al. [29]	LCT	Provide understandings for practitioners	Establish a trust framework
Zhang et al. [30]	CCDP	Maximize total profits	Stochastic plant-pollinator algorithm
Guedes and Borenstein [31]	MDVTRSP	Minimize total transportation costs	Heuristic solution method
Chen et al. [22]	CVRP-RS	Minimize total costs	Extended ant colony optimization

Clustering algorithms are often employed before solving the vehicle routing problem to reduce computational complexity [32]. To solve MDVRP, Yücenur and Demirel [3] proposed a genetic algorithm based on clustering and indicated that the clustering component provided good performance. Reed et al. [33] used k-means clustering to cluster nodes and optimize the logistics network, which improved the efficiency of obtaining optimal solutions. Gao et al. [34] employed a k-means algorithm to solve the location of depots and surrounding cities for a location allocation problem. Defryn and Sörensen [35] grouped customers into multiple clusters; by doing so, vehicles could be reasonably allocated to each cluster. Praveen et al. [36] proposed a new clustering algorithm for effectively mining data, which laid the foundation for further optimization of vehicle routes.

Heuristic algorithms are often used to solve the vehicle routing problem [37, 38], parameter selection optimization [39, 40], and multi-objective optimization problems [41–43]. For example, Dondo et al. [44] developed a mixed-integer linear programming model to solve a large-scale MDVRPTW and then utilized an improved hybrid local search algorithm to obtain feasible routes. Ferdinand et al. [45] presented a heuristic genetic algorithm to solve the pickup and delivery problem with consideration of resource sharing among different logistics providers. Wang and Kopfer [46] connected multiple carriers so that they could respond to different transportation requests, and a heuristic algorithm was presented to solve the centralized vehicle routing problem. Bae and Moon [28] proposed a heuristic algorithm and a genetic algorithm for MDVRPTW, which aimed to minimize the fixed and travel costs of depots. Li et al. [7] proposed an improved ant colony optimization algorithm to solve the MDVRP. Lv et al. [41] proposed a surrogate-assisted particle swarm optimization (PSO) algorithm with Pareto active learning for multi-objective optimization problems. Sedighizadeh and Mazaheripour [42] presented a hybrid algorithm combing the particle swarm optimization and the artificial bee colony algorithms to address multi-objective vehicle routing problems.

### Profit allocation in collaborative logistics networks

In a collaborative logistics network, establishing a collaborative mechanism is critical. Benefits arising from collaboration need to be reasonably split among participants. A fair profit allocation strategy is important for the stability of the logistics network. Cruijssen [47] first proposed the idea of supplier-initiated logistics operation to coordinate shippers and achieve equalities. Dai and Chen [48] addressed two issues in a collaborative logistics network, namely sharing of service requests and profit allocation, and compared the performance of different profit allocation mechanisms. Kumoi and Matsubayashi [49] proposed a cooperative game to analyze stable and fair profit allocations normatively to fairly allocate the profit of a grand coalition. A method based on cooperative game theory has been used to allocate additional profit and promote the participation of consumers in a distributed energy network [50]. Yu et al. [51] calculated the exact Shapley value to distribute profit generated in a collaborative pickup and delivery network. A game theoretic approach has been developed to maximize productivity while ensuring fair profit allocation in collaborative multi-echelon supply chains [52]. Wang et al. [53] proposed a cooperative strategy to minimize carbon emissions in pickup and delivery processes and designed a fair method of profit distribution based on cooperative game theory to stabilize alliances.

In comparison with the aforementioned studies in the domain, the main contributions of this paper are listed in the following aspects: (1) it considers different types of resource sharing schemes, including sharing both within the same DC and across DCs to optimize a collaborative multicenter delivery network; (2) it establishes a bi-objective optimization model for the total operational cost and number of vehicles minimization based on resource sharing in multiple service periods; (3) it employs an AGPSO based on k-means customer clustering, which incorporates an efficient selective endowment mechanism and thus performs well in global and local research; and (4) it implements a real-world case study to evaluate the applicability of the proposed CMCLDN-RS model and approach, which contributes empirically to the literature on collaborative multi-echelon multi-period logistics network optimization, and then lays a foundation for the construction and sustainable development of intelligent transport systems.

### Problem statement

**Fig 1:**
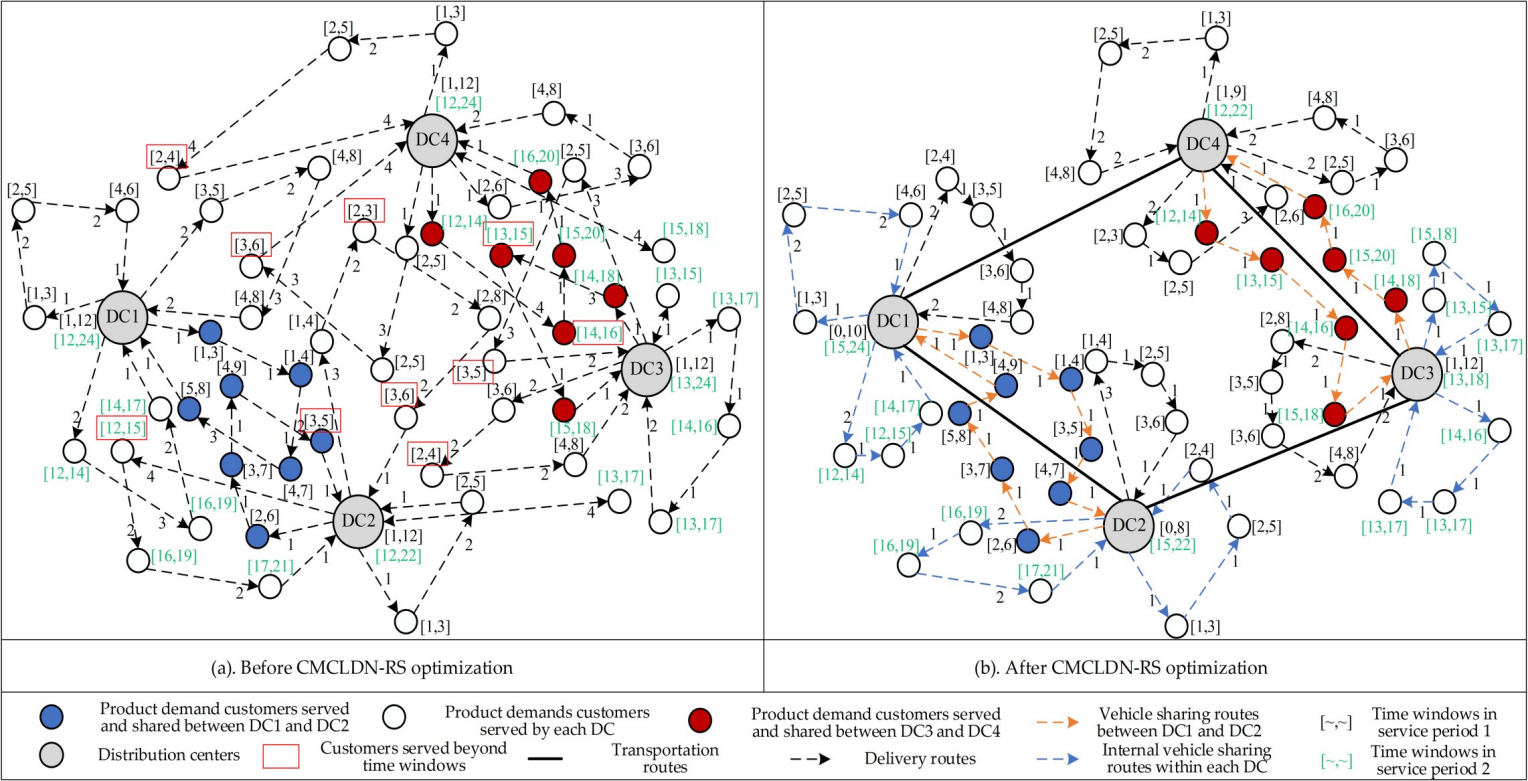
Illustration of the CMLDN-RS problem.

The optimization of CMLDN can improve operational efficiency by using various resource sharing methods [54]. Fig 1 shows the changes in a logistics delivery network when logistics facilities agree to cooperate to achieve resource sharing. Customers can be redistributed among DCs based on the required time, type of service, and geographical locations for cooperative purposes to utilize resources. Transportation between DCs is accomplished by trucks, and vehicles are utilized to serve customers.

As shown in Fig 1A, before CMCLDN-RS optimization, the independent operation of DCs causes serious problems. Primarily, a large number of vehicles for delivery are used due to the different time demands of different customers. In addition, long-distance and cross deliveries not only make the network complex but also increase the delivery cost. These problems significantly reduce the delivery efficiency of the logistics network as a whole. Furthermore, the attributes of some products make them inconvenient to be delivered with other products, which causes a high cost for delivery. In Fig 1B, after CMCLDN-RS optimization, logistics facility resources, vehicle resources, and customer services are shared during the delivery process. Two types of resource sharing schemes are considered: internal vehicle sharing among different service periods, and vehicle sharing among DCs. In the optimized network, not only long-distance and cross deliveries are avoided, but network complexity is also reduced. With the assumption of $30 per unit time for delivery cost and $20 per unit time for penalty cost (earliness and delay penalties), Table 2 shows a comparison of non-collaborative and collaborative cases with the corresponding cost, number of vehicles, and service waiting times.

**Table 2:** Comparison before and after the CMCLDN-RS optimization.

Case	Period	Transportation cost ($)	Delivery cost ($)	Penalty cost ($)	Total cost ($)	Number of vehicles	Service waiting time
Before CMCLDN-RS optimization	1^st^ period	0	2610	380	2990	11	19
	2^nd^ period	0	1350	40	1390	7	2
After CMCLDN-RS optimization	1^st^ period	143	1830	0	1830	11	0
	2^nd^ period	102	840	0	840		0

Table 2 indicates that the central transportation cost, which is $143 in the first service period and $102 in the second, should be considered after CMCLDN-RS optimization. The delivery costs in the first and second service periods are $2610 and $1350 before CMCLDN-RS optimization and $1830 and $840 after, respectively. The total number of vehicles used for serving customers is 18 before CMCLDN-RS optimization and 11 after. The total service waiting time changes from 21 mins to 0, which is a reduction of 21 mins. The results show an obvious reduction in the total cost (from $4380 to $2915) and the number of vehicles (from 18 to 11) in the collaborative logistics network. That is, utilizing resource sharing can reduce the operational costs of the entire delivery network.

### Model formulation

Two resource sharing schemes are considered in the optimization problem of CMCLDN-RS efficiently utilizing logistics resources. Trucks are shared between DCs, and shared vehicles are utilized to serve customers. To make CMCLDN-RS more realistic, several assumptions are illustrated as follows.

Assumption 1: The demands of each customer are known and stable within each service period.
Assumption 2: In the original logistics network, each DC operates independently.
Assumption 3: Each DC pursues maximum profits.

To formulate the CMCLDN-RS optimization problem into a mathematical model, the definitions used in the model are provided in Table 3.

**Table 3:** Symbols and description.

Set	Definition
*K*	Set of trucks for transportation among DCs.
*V*	Set of delivery vehicles for delivery from DCs to customers.
*D*	Set of DCs.
*B*	Set of paired DCs for resource sharing in open delivery.
{*α*,*β*}	Paired DCs, {*α*,*β*}∈*B*.
*C*	Set of customers; each customer can be served via internal vehicle sharing within each DC.
*S*	Set of customers who can be served via vehicle sharing among DCs
Π	Set of service periods, Π = {1,2,3,…,*π*}.
*DK*_π_	Set of trucks for serving DCs within the *π*th service period, *π*∈Π.
*CV*_*π*_	Set of vehicles for visiting customers served via internal vehicle sharing within each DC for the *π*th service period, *π*∈Π.
*SV*_π_	Set of vehicles for visiting customers served via vehicle sharing among DCs within the *π*th service period, *π*∈Π.
**Parameters**	
*c*_*πdcv*_	Demand-dependent cost of delivering goods with vehicle *v* from node *d* to node *c* within the *π*th service period.
*c*_*πidk*_	Demand-dependent cost of transporting goods with truck *k* from distribution *i* to *d* within the *π*th service period.
*c*_*πdsv*_	Demand-dependent cost of delivering goods with vehicle *v* from node *d* to node *s* within the *π*th service period.
|*DK*_*π*_|	Number of trucks for serving DCs within the *π*th service period, *π*∈Π.
|*CV*_*π*_|	Number of delivery vehicles for visiting customers served via internal vehicle sharing within each DC for the *π*th service period, *π*∈Π.
|*SV*_*π*_|	Number of delivery vehicles for visiting customers served via vehicle sharing among DCs within the *π*th service period, *π*∈Π.
|*CN*_*v*_|	Number of customers served by vehicle *v* via internal vehicle sharing.
|*SN*_*v*_|	Number of customers served by vehicle *v* via vehicle sharing among DCs.
*G*_*dπ*_	Subsidies provided to DC *d* if it agrees to cooperate in the two-echelon logistics network within the *π*th service period, *d*∈*D*,*π*∈Π.
*L*_*k*_	Loading capacity of truck *k*.
*L*_*v*_	Loading capacity of delivery vehicle *v*.
*M*_*k*_	Annual maintenance cost of truck *k*.
*M*_*v*_	Annual maintenance cost of delivery vehicle *v*.
*θ*_1_	Penalty cost coefficient for arriving early.
*θ*_2_	Penalty cost coefficient of arriving late.
$Q_{c}^{\pi }$	Demand of customer *c* within the *π*th service period, *c*∈*C*.
$Q_{s}^{\pi }$	Demand of customer *s* within the *π*th service period, *s*∈*S*.
*Q*_*id*_	Delivery quantity from DC *i* to *d*
*MM*	Very large number.
$\left[e_{d}^{\pi },t_{d}^{\pi }\right]$	Operational time window for DC *d* within the *π*th service period, *π*∈Π.
$\left[e_{c}^{\pi },t_{c}^{\pi }\right]$	Service time window for customer *c* within the *π*th service period, *π*∈Π.
$\left[el_{c}^{\pi },ll_{c}^{\pi }\right]$	Acceptable delivery time windows for customer *c* within the *π*th service period.
$at_{d\pi }^{k}$	Truck *k*’s arrival time at node *d* within the *π*th service period, *d*∈*D*∪*C*.
$at_{c\pi }^{v}$	Vehicle *v*’s arrival time at node *c* within the *π*th service period, *c*∈*D*∪*C*.
$at_{s\pi }^{v}$	Vehicle *v*’s arrival time at node *s* within the *π*th service period, *s*∈*D*∪*S*.
$tt_{dcv}^{\pi }$	Vehicle *v*’s travel time from node *d* to node *c* within the *π*th service period, *c*∈*D*∪*C*.
$tt_{dsv}^{\pi }$	Vehicle *v*’s travel time from node *d* to node *s* within the *π*th service period, *s*∈*D*∪*S*.
*F*_*d*_	Fixed cost of DC *d*, *d*∈*D*.
$\left|NR_{v}^{\pi }\right|$	Number of delivery routes within the *π*th service period, *v*∈*V*,*π*∈Π.
**Decision variables**	
*x*_*πidk*_	If truck *k* travels from DC *i* to DC *d* within the *π*th customer service period, then *x*_*πidk*_ = 1; otherwise, *x*_*πidk*_ = 0, *d*∈*D*,*c*∈*D*∪*C*,*v*∈*V*,*π*∈Π.
*x*_*πicdk*_	If the service of customer *c* is changed from DC *i* to DC *d* within the *π*th customer service period, then *x*_*πicdk*_ = 1, otherwise, *x*_*πicdk*_ = 0, *i*,*d*∈*D*,*c*∈*C*,*v*∈*V*,*π*∈Π.
*x*_*πdcv*_	If delivery vehicle *v* travels from node *d* to node *c* within the *π*th customer service period, then *x*_*πdcv*_ = 1, otherwise, *x*_*πdcv*_ = 0, *d*∈*D*∪*C*,*c*∈*D*∪*C*,*v*∈*V*,*π*∈Π.
*x*_*πdsv*_	If delivery vehicle *v* travels from node *d* to node *s* within the *π*th customer service period, *x*_*πdsv*_ = 1, otherwise, *x*_*πdsv*_ = 0, *d*∈*D*∪*C*,*c*∈*D*∪*C*,*v*∈*V*,*π*∈Π.
$\xi_{v}^{\pi }$	If delivery vehicle v is used within the *π*th customer service period, then $\xi_{v}^{\pi }=1$, otherwise, $\xi_{v}^{\pi }=0$, *v*∈*V*,*π*∈Π.
*y*_*dπ*_	If DC *d* agrees to cooperate in vehicle routing optimization within the *π*th customer service period, then *y*_*dπ*_ = 1, *d*∈*D*,*π*∈Π, otherwise, *y*_*dπ*_ = 0.

The CMCLDN-RS optimization model aims to minimize the total operational cost and number of vehicles of a collaborative multicenter delivery network, as shown in Eqs (1) and (2).

$$\min TC=TC_{1}+TC_{2}+TC_{3}$$ (1)

$$\min V=\underset{\pi \in \Pi }{\max}\left\{\min\sum_{v\in V}\xi_{v}^{\pi }\cdot \left(\min\left\{\left|NR_{v}^{\pi }\right|,1\right\}\right)\right\}$$ (2)

*TC*_1_ is the cost for goods transportation. *c*_*πidk*_*x*_*πidk*_ is the transportation cost among DCs, $\underset{\pi \in \Pi }{\max}\left\{\left|DK_{\pi }\right|\right\}\cdot \frac{M_{K}}{T}$ is the truck maintenance cost, *G*_*dπ*_*y*_*dπ*_ refers to the government subsidies when DC *d* joins an alliance, and $\theta_{1}\cdot \max\left\{e_{d}^{\pi }-at_{d\pi }^{k},0\right\}$ and $\theta_{2}\cdot \max\left\{at_{d\pi }^{k}-t_{d}^{\pi },0\right\}$ are the penalty costs for trucks.

$$\begin{array}{c} {TC}_{1}=\sum_{\pi \in \Pi }\sum_{i\in D}\sum_{d\in D,i\neq d}\sum_{k\in K}\left(c_{\pi idk}x_{\pi idk}\right)+\underset{\pi \in \Pi }{\max}\left\{\left|DK_{\pi }\right|\right\}\cdot \frac{M_{K}}{T}\&+\sum_{d\in D}F_{d}-\sum_{d\in D}\sum_{\pi \in \Pi }G_{d\pi }y_{d\pi }\\ +\sum_{k\in K}\sum_{d\in D}\sum_{\pi \in \Pi }\theta_{1}\cdot \max\left\{e_{d}^{\pi }-at_{d\pi }^{k},0\right\}+\sum_{k\in K}\sum_{d\in D}\sum_{\pi \in \Pi }\theta_{2}\cdot \max\left\{at_{d\pi }^{k}-t_{d}^{\pi },0\right\} \end{array}$$ (3)

*TC*_2_ is the cost for goods delivery within each DC. *c*_*πdcv*_*x*_*πdcv*_ is the delivery cost for visiting customers served via closed delivery routes only by one DC, $\theta_{1}\cdot \max\left\{e_{c}^{\pi }-at_{c\pi }^{v},0\right\}$ and $\theta_{2}\cdot \max\left\{at_{c\pi }^{v}-t_{c}^{\pi },0\right\}$ are the penalty costs for vehicles in closed delivery routes, and $\underset{\pi \in \Pi }{\max}\left\{\left|CV_{\pi }\right|\right\}\cdot \frac{M_{v}}{T}$ is the vehicle maintenance cost.

$$\begin{array}{c} {TC}_{2}=\sum_{\pi \in \Pi }\sum_{d\in D\cup C}\sum_{c\in C\cup D}\sum_{v\in V}c_{\pi dcv}x_{\pi dcv}+\sum_{v\in V}\sum_{c\in C}\sum_{\pi \in \Pi }\theta_{1}\cdot \max\left\{e_{c}^{\pi }-at_{c\pi }^{v},0\right\}\\ +\sum_{v\in V}\sum_{c\in C}\sum_{\pi \in \Pi }\theta_{2}\cdot \max\left\{at_{c\pi }^{v}-t_{c}^{\pi },0\right\}+\underset{\pi \in \Pi }{\max}\left\{\left|CV_{\pi }\right|\right\}\cdot \frac{M_{v}}{T} \end{array}$$ (4))

*TC*_3_ is the cost for goods delivery among DCs. *c*_*πdsv*_*x*_*πdsv*_ is the delivery cost for visiting customers served via open delivery routes among DCs, $\theta_{1}\cdot \max\left\{e_{s}^{\pi }-at_{s\pi }^{v},0\right\}$ and $\theta_{2}\cdot \max\left\{at_{s\pi }^{v}-t_{s}^{\pi },0\right\}$ are the penalty costs for vehicles in open delivery, and $\underset{\pi \in \Pi }{\max}\left\{\left|SV_{\pi }\right|\right\}\cdot \frac{M_{v}}{T}$ is the vehicle maintenance cost.

$$\begin{array}{c} {TC}_{3}=\sum_{\pi \in \Pi }\sum_{\begin{array}{l} d\in \gamma \cup S,\\ \gamma \in \left\{\alpha ,\beta \right\} \end{array}}\sum_{\begin{array}{l} s\in \delta \cup S,\\ \delta \in \left\{\alpha ,\beta \right\},\\ \gamma \neq \delta ,d\neq s \end{array}}\sum_{v\in V}c_{\pi dsv}x_{\pi dsv}+\sum_{v\in V}\sum_{s\in S}\sum_{\pi \in \Pi }\theta_{1}\cdot \max\left\{e_{s}^{\pi }-at_{s\pi }^{v},0\right\}\\ +\sum_{v\in V}\sum_{s\in S}\sum_{\pi \in \Pi }\theta_{2}\cdot \max\left\{at_{s\pi }^{v}-t_{s}^{\pi },0\right\}+\underset{\pi \in \Pi }{\max}\left\{\left|SV_{\pi }\right|\right\}\cdot \frac{M_{v}}{T} \end{array}$$ (5)

Subject to:

$$\sum_{v\in V}\sum_{d\in D\cup C}x_{\pi dcv}=1,c\in C,\pi \in \Pi$$ (6)

$$\sum_{d\in D\cup S}\sum_{v\in V}x_{\pi dsv}=1,\pi \in \Pi ,s\in S$$ (7)

$$\sum_{c\in C}Q_{c}^{\pi }x_{\pi dcv}\leq L_{v},d\in D,v\in V,\pi \in \Pi$$ (8)

$$\sum_{s\in S}Q_{s}^{\pi }x_{\pi dsv}\leq L_{v},v\in V,d\in \left\{\alpha ,\beta \right\},\pi \in \Pi$$ (9)

$$\sum_{d\in D\cup C}x_{\pi dcv}-\sum_{j\in D\cup C}x_{\pi cjv}=0,c\in C,v\in V,\pi \in \Pi$$ (10)

$$\sum_{d,c\in C}x_{\pi dcv}\leq |CN_{v}|-1,v\in V,\pi \in \Pi$$ (11)

$$\sum_{d,s\in S}x_{\pi dsv}\leq |SN_{v}|-1,v\in V,\pi \in \Pi$$ (12)

$$\sum_{c\in C}\sum_{v\in V}Q_{c}^{\pi }x_{\pi dcv}+\sum_{s\in S}\sum_{v\in V}Q_{s}^{\pi }x_{\pi dsv}\leq Q_{d}^{\pi },\pi \in \Pi ,d\in D$$ (13)

$$\sum_{c\in C}Q_{c}^{\pi }x_{\pi icdk}=Q_{id},i,d\in D,k\in K$$ (14)

$$\sum_{s\in S}Q_{s}^{\pi }x_{\pi isdk}=Q_{id},i,d\in D,k\in K$$ (15)

$$dt_{d\pi }^{v}+tt_{dcv}^{\pi }-MM\left(1-x_{\pi dcv}\right)\leq at_{c\pi }^{v},c,d\in D\cup C,v\in V,\pi \in \Pi$$ (16)

$$dt_{d\pi }^{v}+tt_{dcv}^{\pi }+MM\left(1-x_{\pi dcv}\right)\geq at_{c\pi }^{v},c,d\in D\cup C,v\in V,\pi \in \Pi$$ (17)

$$dt_{d\pi }^{v}+tt_{dsv}^{\pi }-MM\left(1-x_{\pi dsv}\right)\leq at_{s\pi }^{v},c,d\in D\cup S,v\in V,\pi \in \Pi$$ (18)

$$dt_{d\pi }^{v}+tt_{dsv}^{\pi }-MM\left(1-x_{\pi dsv}\right)\leq at_{s\pi }^{v},c,d\in D\cup S,v\in V,\pi \in \Pi$$ (19)

$$dt_{d\pi }^{v}+tt_{dcv}^{\pi }\leq ll_{c}^{\pi },c,d\in D\cup C,v\in V,\pi \in \Pi$$ (20)

$$dt_{d\pi }^{v}+tt_{dcv}^{\pi }\geq el_{c}^{\pi },c,d\in D\cup C,v\in V,\pi \in \Pi$$ (21)

$$x_{\pi icd}=\left\{0,1\right\},i,c,d\in C\cup D,\pi \in \Pi$$ (22)

$$x_{\pi dcv}=\left\{0,1\right\},d\in D,c\in D\cup C,v\in V,\pi \in \Pi$$ (23)

$$x_{\pi dsv}=\left\{0,1\right\},d\in D,s\in D\cup S,v\in V,\pi \in \Pi$$ (24)

$$x_{\pi icdk}=\left\{0,1\right\},i,d\in D,c\in C,v\in V,\pi \in \Pi$$ (25)

$$y_{d\pi }=\left\{0,1\right\},d\in D,\pi \in \Pi$$ (26)

Constraints (6) and (7) ensure that each customer is served by only one DC. Constraints (8) and (9) guarantee that the delivery loads do not exceed the vehicle capacity. Constraint (10) ensures the conservation of goods flow for visiting customers served via internal vehicle sharing within each DC. Constraints (11) and (12) aim to eliminate the subtour in the delivery process. Constraint (13) guarantees that total customer demands do not exceed the DC capacity. Constraints (14) and (15) stipulate the delivery quantity from DC *i* to *d*, which is equivalent to the total change of customer quantities from DC *i* to *d*. Constraints (16) and (17) limit the arrival time of each vehicle for visiting customers served by internal vehicle sharing within each DC. Constraints (18) and (19) limit the arrival time of each vehicle for visiting customers served by vehicle sharing among DCs. Constraints (20) and (21) guarantee that each customer is served in their expected time windows. Constraints (22)–(26) are binary decision variables.

## Research methodologies

### Related definitions and solution procedure

**Fig 2:**
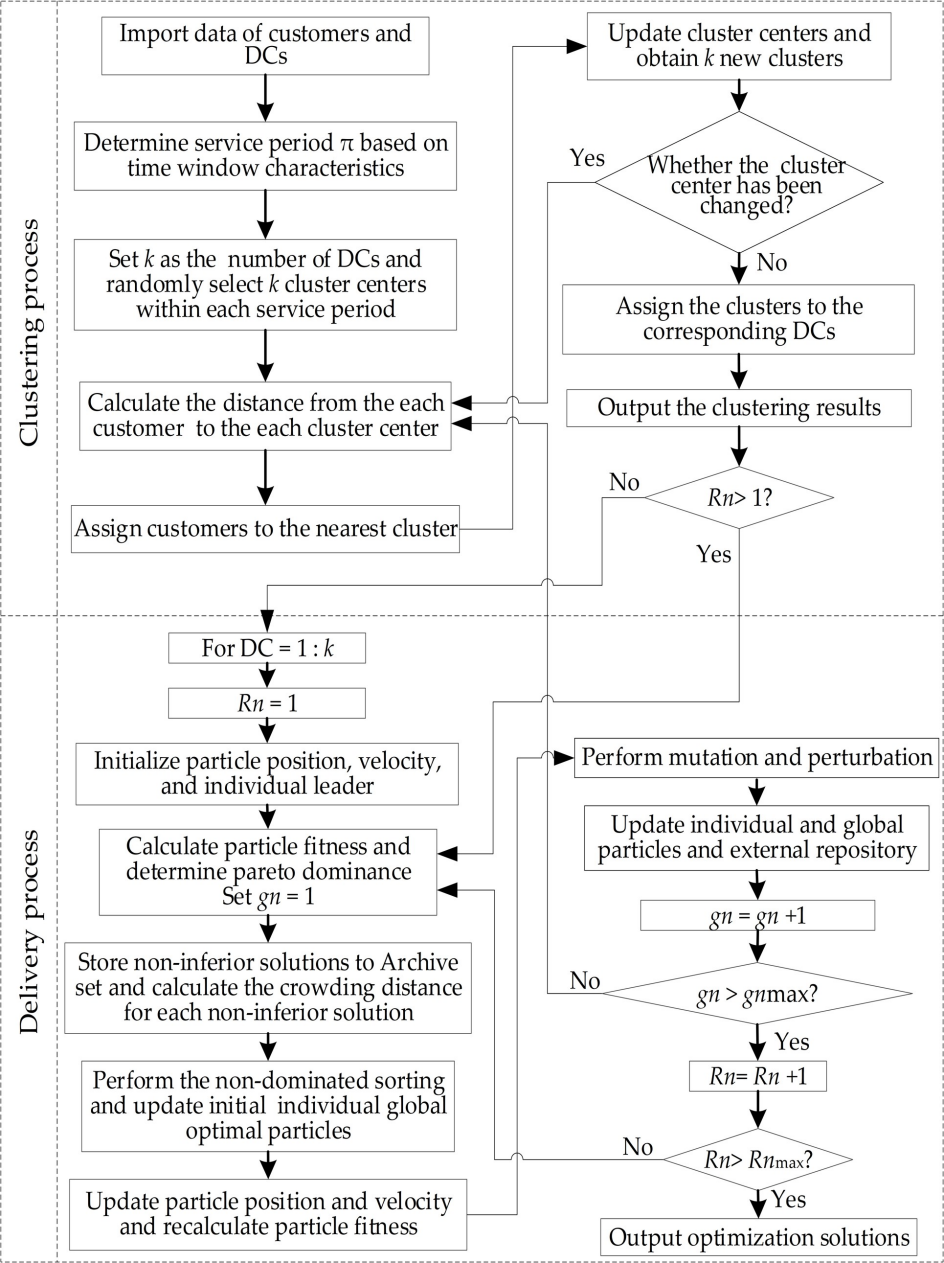
AGPSO flowchart.

Our proposed CMCLDN-RS problem not only can improve the operational efficiency of a multicenter distribution network but can also reduce the network operation costs. Fig 2 shows the AGPSO process for solving the CMCLDN-RS problem. To clarify the relevant optimization process, several required parameters are defined as follows.

*nPOP*: Population size
*nREP*: Repository size
*gn*max: Maximum number of iterations
*Rn*max: Maximum number of optimization runs
*W*: Coefficient of inertia
*R*_1_: Personal learning coefficient
*R*_2_: Global learning coefficient
*nGrid*: Number of grids per dimension
*α*: Inflation rate
*β*: Leader selection pressure
*γ*: Deletion selection pressure
*Mut*: Mutation rate

### Adaptive grid particle swarm optimization

Due to the complexity of CMCLDN-RS when considering a large number of customers, it is inefficient to solve the heuristic algorithm directly. Therefore, the combination of a clustering algorithm and AGPSO is used to solve the CMCLDN-RS optimization problem. AGPSO aims to make the obtained optimal Pareto solution as close as possible to the true Pareto optimal frontier. The Pareto solutions are calculated based on the nondominated sorting and crowding distance for each generation, and then the optimal Pareto is filtered out of all the Pareto solutions [55, 56]. First, the nondominated solutions are sorted in accordance with the value of the bi-objective function, and the nondominated solutions in the Pareto frontier with peer rank has the same degree of optimization. Second, the crowding distance of each particle is calculated as the sum of the distance values that correspond to each objective for every particle. Third, the Pareto frontier of different ranks are obtained. Finally, the optimal non-dominated solutions can be considered and selected as the solution with peer rank based on the focus of the bi-objective.

The most important components of AGPSO are determining how to choose the personal best position *pbest* and global best position *gbest* and how to maintain the external repository. For AGPSO, the method to select the best position of an individual is to compare the current particle position with the best historical position of the individual. If the new solution dominates the current *pbest*, then the new solution acts as the new *pbest*. *gbest* is not unique in multi-objective optimization, presenting multiple global optimal solutions that are not dominated by one another. These solutions are stored in the external repository. The external repository not only stores the nondominated vectors obtained along the search process but also plays a role in guiding the population to the latest Pareto frontier. In addition, the Pareto frontier can be extended to the Pareto front surface to select the optimal solution for solving problems involving more than two objective functions [57]. The specific process of AGPSO is explained as follows, and the pseudo-code procedure is shown in Table 4.

1. Customer clustering: The imported customer data are clustered based on the attributes of customers. Manhattan distance is utilized to calculate the distance between customers.
2. Population initialization: Population *POP* and the velocity of each particle are initialized, and the initial vehicle routes are generated according to the capacity constraints (8)-(9), subtour constraints (11)-(12), and time window constraints (16)-(21), and then the objective function values corresponding to each particle are found. The locations that replace the nondominated vectors are stored in the repository *REP*.
3. Fitness function value calculation: The fitness function values include the total operational cost in Eq (1) and number of vehicles in Eq (2), and the two objectives constitute a two-dimensional coordinate, which is used to obtain the Pareto frontier of different ranks based on the nondominated sorting and crowding distance. The fitness function values of each particle are calculated based on the crowding distance of each particle and the nondominated sorting process. The initial personal best position *pbest* and global best position *gbest* of the particle are determined on the basis of the population initialization scheme.
4. Velocity and position update and *pbest* adjustment: *W* is the inertia weight that controls the convergence of the algorithm, and *R*_1_ and *R*_2_ are uniform random numbers in interval [0,1]. If *pbest* is dominated by the new particle position, then *pbest* will be replaced by the new position. If any one is not dominated by others, then one with a probability is randomly selected.
5. External repository maintenance: The objective function values of each particle in the newly generated population *P*_*t+1*_ is calculated, when the accumulated demands exceed the capacity of the vehicle according to the capacity constraints (8), (9) and (13), in addition, it will return to the departure DC for the internal vehicle sharing routes, for vehicle sharing between DCs, it will return to an adjacent DC for the vehicle sharing routes among DCs. The positions that replace the nondominated vectors are stored in repository *REP*. The external repository is maintained and updated, and *REP*_*t+1*_ is generated, and then *gbest* of each particle is chosen.
6. Algorithm termination conditions: If the algorithm reaches the termination condition then the iteration is stopped; otherwise, *t = t +* 1, and step (3) is repeated. When the algorithm terminates, the current external repository *A*_*t*_ is the Pareto solution set.

**Table 4:** AGPSO algorithm procedure.

AGPSO algorithm:
**Input**: customers’ corresponding data, POP(population size), *REP*(external repository), *It*max(maximum number of iterations)
**Output:** Pareto-optimal solutions.
**Step 1:** Define *o* as the number of clusters.
**Step 2:** Randomly choose *o* original cluster centers.
Repeat Steps 3 and 4 until the membership in each cluster becomes stable.
**Step 3:** (Re-)assign each customer to a cluster whose cluster center is the closest.
**Step 4:** Update the center of each cluster.
**Step 5:** Output the clustering results.
**Step 6:** Initialize the population *POP*[*i*] and the velocity of each particle VEL[*i*].
Evaluate each of the particles in *POP*
**Step 7:** Calculate the objective function value of each particle.
Store the non-dominated vectors in *REP*.
**while *i***≤*It_max_*
**do**
Update the velocity of each particle:
*VEL*[*i*+1]≤*W*×*VEL*[*i*]+*R*_1_×{*PBESTS*[*i*]−*POP*[*i*]}+*R*_2_×{*PBESTS*[*h*]−*POP*[*i*]}
Calculate the new position of the particle: *POP*[*i*+1] = *POP*[*i*]+*VEL*[*i*]
Maintain the particles within the search space
Evaluate each of the particles in *POP*
Update the repository together with the geographical representation of the hypercubes
Update the particle position: *PBESTS*[*i*] = *POP*[*i*]
**end while**

#### Adaptive grid density estimation algorithm

An adaptive grid density estimation algorithm is utilized to update the external repository. The updates of the external repository must meet either of the following two conditions: (1) the newly-generated particles dominate one or more particles in the external repository; (2) the number of particles in the external repository has reached the allowed capacity. The adaptive grid algorithm first appeared in the Pareto archived evolution strategy [56, 58]. The adaptive grid density estimation algorithm needs to compute the density information of particles in *Archive*. The target space is divided into small cells with a grid and the number of particles contained in each area is used as the density information of the particles. The main procedure is as follows.

Step 1: The boundary of the target space during *t*-generation evolution $\left(\min F_{1}^{t},\max F_{1}^{t}\right)$ and $\left(\min F_{2}^{t},\max F_{2}^{t}\right)$ is calculated.
Step 2: The Modulus of the grid is calculated as follows: $\Delta F_{1}^{t}=\frac{\max F_{1}^{t}-\min F_{1}^{t}}{M};\Delta F_{2}^{t}=\frac{\max F_{2}^{t}-\min F_{2}^{t}}{M}$.
Step 3: The indices of the grids in which particles are calculated. For particle *i*, the grid index consists of two parts: $\left(Int(\frac{F_{1}^{i}-\min F_{1}^{i}}{\Delta F_{1}^{t}})+1,Int(\frac{F_{2}^{i}-\min F_{2}^{i}}{\Delta F_{2}^{t}})+1\right)$.
Step 4: Grid information is calculated and the number of particles in the grid is saved to array *Grid* [].
Step 5: Particle density estimates are calculated and the results are stored to array *Archive_Obj*[].

#### Archive truncation operation

When the number of particles in the archive exceeds the allowed capacity, excess individuals need to be discarded to maintain the stability of the archive. For grid *g* with more than one particle, the number of particles *PN* to be deleted in the grid *g* is calculated using Eq (27), then *PN* particles are randomly discarded in it.

$$PN=Int\left(\frac{|A_{t+1}|-\bar{N}}{\left|A_{t+1}\right|}\times Grid[g,2]+0.5\right)$$ (27)

**Fig 3:**
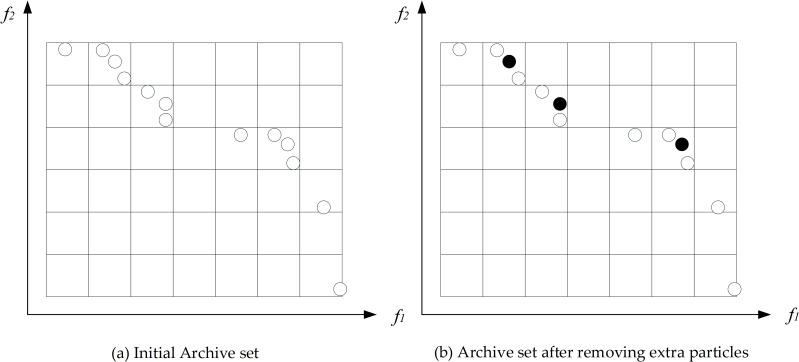
Illustration of the archive truncation operation.

*Grid* [*g*] represents the number of contained particles in grid *g*. The schematic of the archive truncation operation is shown in Fig 3. The black circles in Fig 3B are the deleted particles.

#### Mutation operator

To avoid the PSO-based algorithm to converge to a false Pareto front, Coello et al. [59] proposed a new mutation operator in MOPSO, which considers the effect of the mutation operator on the number of iterations and particle swarms. The mutation operator is used in this paper. Its pseudocode is illustrated in Table 5. *pm* is the particle to be mutated, *dims* is the number of dimensions, *currentgen* is the current iteration, *totgen* is the total number of iterations, and *mr* is the mutation rate.

**Table 5:** Procedure of the mutation operator.

Mutation operator:
1: **function** Mutation Operator (pm, dims, currentgen, totgen, mr)
2: wdim = random (0, dims-1)
3: mutrange = (upperbound[wdim]-lowerbound[wdim])*(1-currengen/totgen)^5/mutrate^
4: ub = particle[wdim]+mutrange
5: lb = particle[wdim]-mutrange
6: **if** lb < lowerbound
7: lb = lowerbound[wdim]
8: **end**
9: **if** ub > upperbound
10: ub = upperbound[wdim]
11: **end**
12: particle[wdim] = RealRandom(lb,ub)
13: **end**

### Profit allocation method

For the CMCLDN-RS optimization problem, collaborative alliances can achieve resource sharing among multiple centers and reduce the total operational cost of collaborative alliances. The cost saving generated by collaboration should be fairly allocated to each participant. MCRS is a game theory method for solving the problem of cost or benefit allocation [4, 60]. We suppose that *N* = {1,2,3,…,*i*} can be the set of all participants and *A* be a subset of *N*. *V*(*N*) is the total profits of the alliance with all participants in *N* and *V*(*A*) is the profit of *A* and can be calculated by Eq (28).

$$V(A)=(1-\sigma )\max\{\sum_{i\in S}C_{0}(i)-C(A),0\},\;A\subseteq N$$ (28)

In Eq (28), *C*_0_(*i*) is the initial cost of participant *i*. *C*(*A*) is the optimized cost of alliance *A*. *σ* is the synergy coefficient of the logistics provider that facilitates the collaboration among DCs. The upper and lower bounds of the profit allocation method are *X*_min_ = {*X*_1min_,⋯,*X*_*j*min_,⋯,*X*_*n*min_} and *X*_max_ = {*X*_1max_,⋯,*X*_*j*max_,⋯,*X*_*n*max_}, respectively. *X*_*j*max_ expresses the maximum profits of DC *j*, and *X*_*j*min_ expresses the minimum profit of DC *j*. The actual profit of DC *j* can be calculated using Eq (29).

$$X_{j}(A,V)=X_{j\min}+\frac{X_{j\max}-X_{j\min}}{\sum_{j\in N}\left(X_{j\max}-X_{j\min}\right)}\times (V(A)-\sum_{j\in N}X_{j\min})$$ (29)

In Eq (29), *X*_*j*max_ and *X*_*j*min_ can be calculated using Eqs (29) and (30) as follows, where *V*(*A*−{*j*}) is the residual profit of alliance *A* except participant *j* and *V*(*j*) is the profit of participant *j*.

$$X_{j\min}=V(j)$$ (30)

$$X_{j\max}=V(A)-V(A-\{j\})$$ (31)

Subject to

$$\sum_{j\in A}X_{j}\geq V(A)$$ (32)

$$\sum_{j\in N}X_{j}=V(N)$$ (33)

$$X_{j}\geq V(j)$$ (34)

$$X_{j\min}\leq X_{j}(A,V)\leq X_{j\max}$$ (35)

Eqs (32) and (34) ensure the collective profit of each alliance and participant. Eq (35) guarantees that the value of *X*_*j*_(*A*,*V*) is between *X*_*j*min_ and *X*_*j*max_. The Cost reduction percentage *η*(*i*,*ϕ*,*u*) of participant *i* in sequence *ϕ* when the *u*th participant joins the alliance is calculated using Eq (36).

$$\eta (i,\phi ,u)=\frac{X_{i}(\cup_{\phi (\mu )\leq u,\mu \in A}\mu ,V)}{C_{0}(i)},\phi (i)\leq \mu$$ (36)

## Case study

### Algorithm comparison

The proposed AGPSO algorithm, nondominated sorting genetic algorithm-II (NSGA-II) [61], and multi-objective evolutionary algorithm (MOEA) [62] are tested using 36 different datasets to evaluate the applicability of the proposed algorithm to CMCLDN-RS optimization. Table 6 shows the data from each of the 36 groups. The total operational cost, the number of vehicles used for delivery, and the computation time are compared to assess the effectiveness of AGPSO. The selection process of related parameters is important and necessary, and there are several methods to select related parameters, including comparative experiment and selection [4, 39, 40, 63, 64], orthogonal experimental design [65], etc. A parameter sensitivity analysis is performed to determine the parameter sensitivity and select parameter values. Through a large number of computational experiments and comparisons, we find that the coefficient of inertia, Inflation rate, and mutation rate are relatively sensitive, and when the coefficient of inertia is set as the medium value, and the inflation rate and the mutation rate are set as small values, the optimal costs and number of vehicles are more likely to be obtained in a short time. The related parameters of AGPSO are illustrated as follows: *nPOP* = 50, *nREP* = 15, *W* = 0.5, *R*_1_ = 1, *R*_2_ = 2, *nGrid* = 7, *α* = 0.1, *β* = 2, *λ* = 2, *mut* = 0.1. In addition, *TS* = 40 represents the travel speed, *L*_*k*_ = 1500, *L*_*v*_ = 200, *M*_*k*_ = 1500, *M*_*v*_ = 500, *θ*_1_ = 0.05, *θ*_2_ = 0.1. The optimal total cost of logistics operation, number of vehicles and computation time are calculated and compared among the three algorithms with 20 randomly generated datasets are shown in Table 7.

**Table 6:** Description of instances.

Instance	Number of customers	Number of DCs
1–4	90	2,4,6,8
5–8	110	2,4,6,8
9–12	130	4,6,8,10
13–16	150	4,6,8,10
17–20	200	6,8,10,12
21–24	240	6,8,10,12
25–28	300	8,10,12,14
29–32	360	10,12,14,16
33–36	400	10,12,14,16

**Table 7:** Comparison of the results of algorithm optimization.

Instance	AGPSO			NSGAII			MOEA		
	Cost	No. of Vehicles	Time (s)	Cost	No. of Vehicles	Time (s)	Cost	No. of Vehicles	Time (s)
	($)			($)			($)		
1	7539	5	202.4	8407	5	209.3	11445	5	223.7
2	8534	6	176.3	8397	6	175.2	8983	6	167.2
3	10734	4	129.3	10279	4	129.3	10432	4	145.2
4	14368	3	92.3	15452	3	85.9	15126	3	84.2
5	9499	8	210.4	10575	8	212	11764	8	214.3
6	10840	5	169.6	11316	5	181.6	12408	6	159.7
7	13393	5	154.2	16600	5	153.8	18387	5	140.3
8	15630	4	98.2	18728	4	94.8	19652	5	91.9
9	11226	10	279.2	11674	10	290.2	12922	11	271.5
10	11230	10	246.2	11870	10	251.8	11859	10	240.8
11	15938	8	201.3	19037	8	203.4	17902	8	179.8
12	22269	6	139.1	24526	6	140.7	25027	7	138.1
13	11788	12	268.9	12659	12	271.4	13342	12	242.7
14	14053	10	236.2	14387	10	234.1	15175	10	221.3
15	21603	9	200.3	24334	9	194.1	25730	9	181.2
16	28731	8	148.3	32892	8	153.8	35883	8	139.7
17	22029	17	366.2	23170	17	374.2	24739	17	357.8
18	20627	15	305.8	21136	15	305.6	22191	15	321.7
19	21180	15	283.2	22596	14	281.9	21739	14	274.5
20	24831	15	251.3	26318	15	250.6	27098	15	246.3
21	25316	18	382.5	26835	19	375.8	27103	19	364.5
22	26122	16	365.4	27195	17	336.7	27876	18	327.3
23	27393	17	343.7	28236	17	348.5	28967	18	335.6
24	27964	17	352.6	29198	18	361.7	29873	18	358.7
25	28231	20	410.2	28972	21	421.6	28772	21	398.2
26	28965	18	393.4	29325	18	410.2	29967	19	401.7
27	29538	18	414.7	30122	19	425.1	30351	19	410.5
28	30419	19	387.3	31028	20	407.5	31516	20	412.1
29	30687	22	433.5	31127	23	418.2	31238	22	406.3
30	31293	21	441.6	32165	22	428.7	32157	22	425.2
31	31572	21	446.2	32381	21	435.4	32764	21	451.6
32	32091	22	455.9	32768	22	462.1	33150	22	442.5
33	31242	23	452.5	31758	24	430.4	32161	23	417.7
34	31894	23	457.3	32641	23	441.6	32985	23	431.6
35	32325	22	462.1	32965	23	438.2	33029	23	435.3
36	32879	22	468.9	33327	23	451.3	34101	23	448.1
Average	22055	14	300.7	23178	14	299.6	23828	14	291.9
*t*-test				-6.70			-7.19		
*p*-value				4.61E-08			1.08E-08		

The best solution and computation time returned by each algorithm for each data instance is listed in Table 7. *t*-test and *p*-value results for optimal logistics operational costs are shown at the bottom of Table 7, which indicates that NSGAII and MOEA are significantly different from AGPSO. Regarding cost optimization, AGPSO performs better than NSGAII and GA-TS in most cases. For example, the average cost of the 36 instances of AGPSO is $22055, whereas NSGAII and GA-TS are $23178 and $23828, respectively. The optimization effectiveness for the number of vehicles is the same amongst all three algorithms. They all have an average number of 14 vehicles. For computation time, the proposed AGPSO tends to take the most time to converge among the three algorithms. MOEA performs well in computation time but is inferior in the cost optimization effectiveness.

### Data description

A practical case of CMCLDN-RS optimization, conducted in Chengdu, China, is studied to test the applicability of the proposed logistics network optimization mechanism. Four DCs (DC1, DC2, DC3, and DC4) and 180 customers (C1, C2, …, C180) are selected from the complex network to demonstrate the effectiveness of CMCLDN-RS optimization. The coordinate location information of four DCs and 180 customers is shown in S1 Dataset. Table 8 shows the characteristics of all DCs and their allocated number of customers. Table 9 shows the initial assignment of customers served by each DC in the initial non-collaborative logistics network.

**Table 8:** Characteristics of logistics facilities.

Facility	Number of allocated customers	Longitude	Latitude	Time windows	
DC1	27	103.954	30.8073	0	900
DC2	29	103.9451	30.56697	0	900
DC3	32	104.4326	30.76488	0	900
DC4	31	104.3035	30.56581	0	900

**Table 9:** Initial assignment of customers served by each DC.

Facility	Customers allocation
DC1	D1 D2 D3 D4 D5 D6 D7 D9 D12 D14 D16 D17 D20 D21 D22 D25 D26 D28 D29 D30 D31 D32 D33 D34 D36 D37 D38 D39 D41 D42 *D8 *D10 *D11 *D13 *D15 *D18 *D19 *D23 *D24 *D27 *D35 *D40
DC2	D43 D46 D48 D50 D53 D54 D56 D57 D58 D60 D61 D62 D64 D65 D66 D67 D68 D69 D70 D71 D72 D73 D74 D75 D76 D78 D79 D80 D83 D84 D86 D88 *D44 *D45 *D47 *D49 *D51 *D52 *D55 *D59 *D63 *D77 *D81 *D82 *D85 *D87 *D89 *D90
DC3	D91 D94 D95 D96 D102 D103 D108 D110 D111 D113 D115 D116 D117 D118 D119 D120 D121 D122 D123 D124 D125 D126 D128 D129 D130 D131 D132 D133 D134 *D92 *D93 *D97 *D98 *D99 *D100 *D101 *D104 *D105 *D106 *D107 *D109 *D112 *D114 *D127
DC4	D135 D136 D138 D139 D141 D143 D144 D147 D148 D149 D150 D151 D152 D154 D158 D159 D160 D161 D162 D164 D165 D166 D167 D168 D169 D170 D171 D172 D174 D175 D176 D177 D178 D179 D180 *D137 *D140 *D142 *D145 *D146 *D153 *D155 *D156 *D157 *D163 *D173

*: Customers potentially shared among DCs.

In Table 9, customers with asterisks are potentially shared among DCs. In addition, Table 9 indicates that the existence of unreasonable customer services such as cross- and long-distance transportations increase the complexity of the logistics network; thus, it’s essential to study the collaboration mechanism and resource sharing schemes for CMCLDN-RS among multiple DCs across several periods.

### Optimization results

As mentioned in the above model formulation, the global sharing for the collaborative logistics network consists of two collaborative modes. One mode consists of internal vehicle sharing within each DC in different service periods. Some products in different DCs have similar but unique attributes and are unsuitable for delivering with others; thus, collaboration among DCs is considered so that vehicle sharing can deliver such category of products. In this study, such a situation exists between DC1 and DC2 and between DC3 and DC4 in the initial multi-center logistics network. Therefore, vehicle sharing between DC1 and DC2 and between DC3 and DC4 should be considered. The other collaborative mode is vehicle sharing among DCs in different service periods (i.e., considering products of DCs with similar attributes but which cannot be delivered with general products).

The parameter sensitivity analysis process is performed to select parameter values in the optimization model as follows. The fixed cost of each DC is: *F*_1_ = 1200, *F*_2_ = 1000, *F*_3_ = 900, *F*_4_ = 1100. Incentives offered to each participant are: *G*_1_ = 298, *G*_2_ = 341, *G*_3_ = 268, *G*_4_ = 314. The parameters used for AGPSO are as follows [39, 40, 63, 64]: *nPOP* = 150, *nREP* = 20, *W* = 0.5, *R*_1_ = 1, *R*_2_ = 2, *nGrid* = 9, *α* = 0.1, *β* = 2, *λ* = 2, *mut* = 0.1, *TS* = 40, *L*_*k*_ = 1500, *L*_*v*_ = 200, *M*_*k*_ = 1500, *M*_*v*_ = 500, *θ*_1_ = 0.05, *θ*_2_ = 0.1. We consider two collaborative forms in this study, and 52 working periods are included in a year. The AGPSO algorithm is used to reassign customers and calculate total cost in a working period. Cost savings generated by optimizing the initial network are allocated via MCRS. Details are discussed below. Table 10 indicates the assignment of customers served via internal vehicle sharing within each service period in the grand alliance. Table 11 shows the assignment of customers served among DCs within each service period.

**Table 10:** Assignment of customers served via internal vehicle sharing within each DC in the grand alliance.

Facility	Customers allocation
DC1	Period 1: D3 D5 D12 D20 D21 D22 D38 D58 D60 D115 D122 D123 D170
	Period 2: D6 D33 D34 D83 D102 D103 D121 D164 D166 D179 D180 D14 D2
	Period 3: D1 D4 D7 D9 D56 D62 D84 D120 D17 D61
DC2	Period 1: D42 D53 D78 D86 D116 D117 D119 D132 D177
	Period 2: D41 D43 D46 D50 D57 D118 D130 D174 D178 D88
	Period 3: D25 D26 D48 D54 D64 D76 D79 D131 D158 D159 D175
DC3	Period 1: D68 D69 D70 D91 D96 D111 D113 D162 D169
	Period 2: D31 D94 D95 D128 D144 D150 D151 D165 D152
	Period 3: D29 D30 D39 D66 D67 D124 D125 D126 D161
DC4	Period 1: D16 D28 D75 D108 D110 D136 D139 D167 D171 D172 D176
	Period 2: D133 D141 D148 D160 D72 D74 D129 D134 D135 D168
	Period 3: D32 D36 D37 D65 D71 D73 D80 D147 D149 D154

**Table 11:** Routes of customers served among DCs.

*A*	Routes among DCs
{DC1, DC2}	Period 1: DC1→D24→D42→D19→D35→D89→D10→DC2→D49→D44→D47→DC2
	Period 2: DC2→D8→D27→D63→D11→D47→D81→DC1→D13→D23→D55→D59→DC1
	Period 3: DC1→D18→D15→D90→DC2→D85→D87→D77→D82→D51→D40→DC2
{DC3, DC4}	Period 1: DC3→D105→D142→D155→D146→DC4→D100→D109→D140→D101→D156→DC4
	Period 2: DC4→D137→D107→D127→D153→DC3→D98→D104→D145→D99→D112→D114→DC3
	Period 3: DC3→D173→D92→D138→D106→D143→DC4→D157→D163→D93→D97→DC4

Table 11 shows the open delivery routes (i.e. sharing among DCs) after CMCLDN-RS optimization. In this sharing mode, the DC1-DC2 pair and DC3-DC4 pair collaborate to achieve vehicle sharing among the three service periods. For example, in period 1 of alliance {DC1, DC2}, the vehicle departs from DC1 and arrives at DC2 after serving D24, D42, D19, D35, D89, and D10, and then departs from DC2 to serve D49, D44, and D47 and finally returns to DC2. The same vehicle departs from DC2 to serve related customers in period 2, which is also shared in period 3.

**Fig 4:**
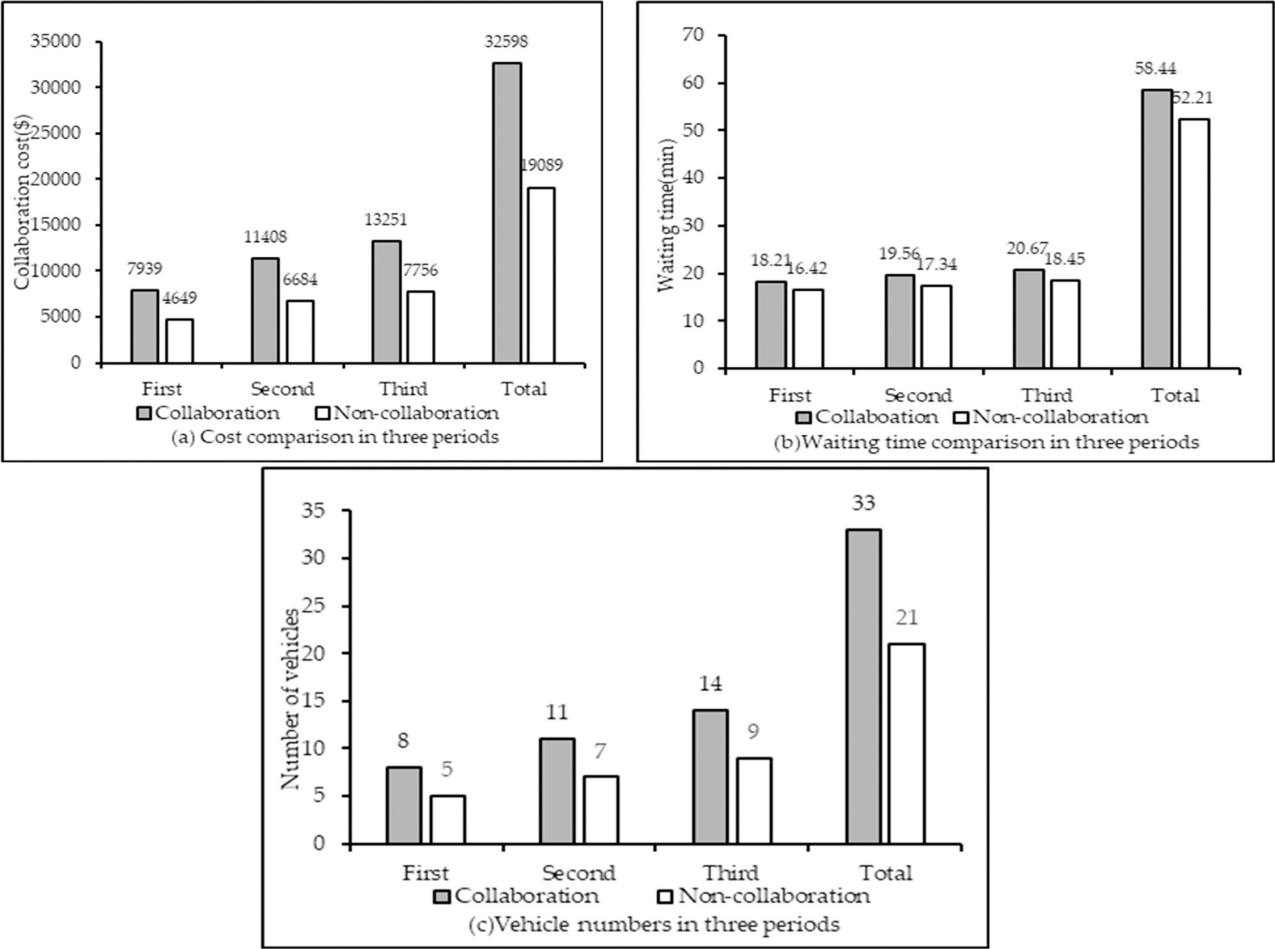
Result comparison before and after CMCLDN-RS optimization in three periods.

For global sharing, the result comparison before and after CMCLDN-RS optimization in three periods is shown in Table 12 and Fig 4. The number of customers to be served and the customer needs in each service period are different; thus, the logistics operational cost, service waiting time, and number of vehicles used in different periods also differ. However, the three components are all reduced after CMCLDN-RS optimization in the same service period. For example, the logistics operational cost decreases from $7939 to $4649, the service waiting time changes from 18.21 min to 16.42 min, and the number of vehicles decreases from 8 to 5 after collaboration in the first service period. From a holistic perspective, the total logistics operational cost, service waiting time, and number of vehicles used for three service periods are all reduced, which indicates that the CMCLDN-RS optimization is effective in coordinating logistics resources.

**Table 12:** Result comparison before and after CMCLDN-RS optimization in three periods.

Period	Logistics operational cost ($)		Service waiting time (min)		Number of vehicles	
	Non-collaboration	Collaboration	Non-collaboration	Collaboration	Non-collaboration	Collaboration
Period 1	7939	4649	18.21	16.42	8	6
Period 2	11408	6684	19.56	17.34	11	9
Period 3	13251	7756	20.67	18.45	14	11
Total	32598	19089	58.44	52.21	33	21

**Fig 5:**
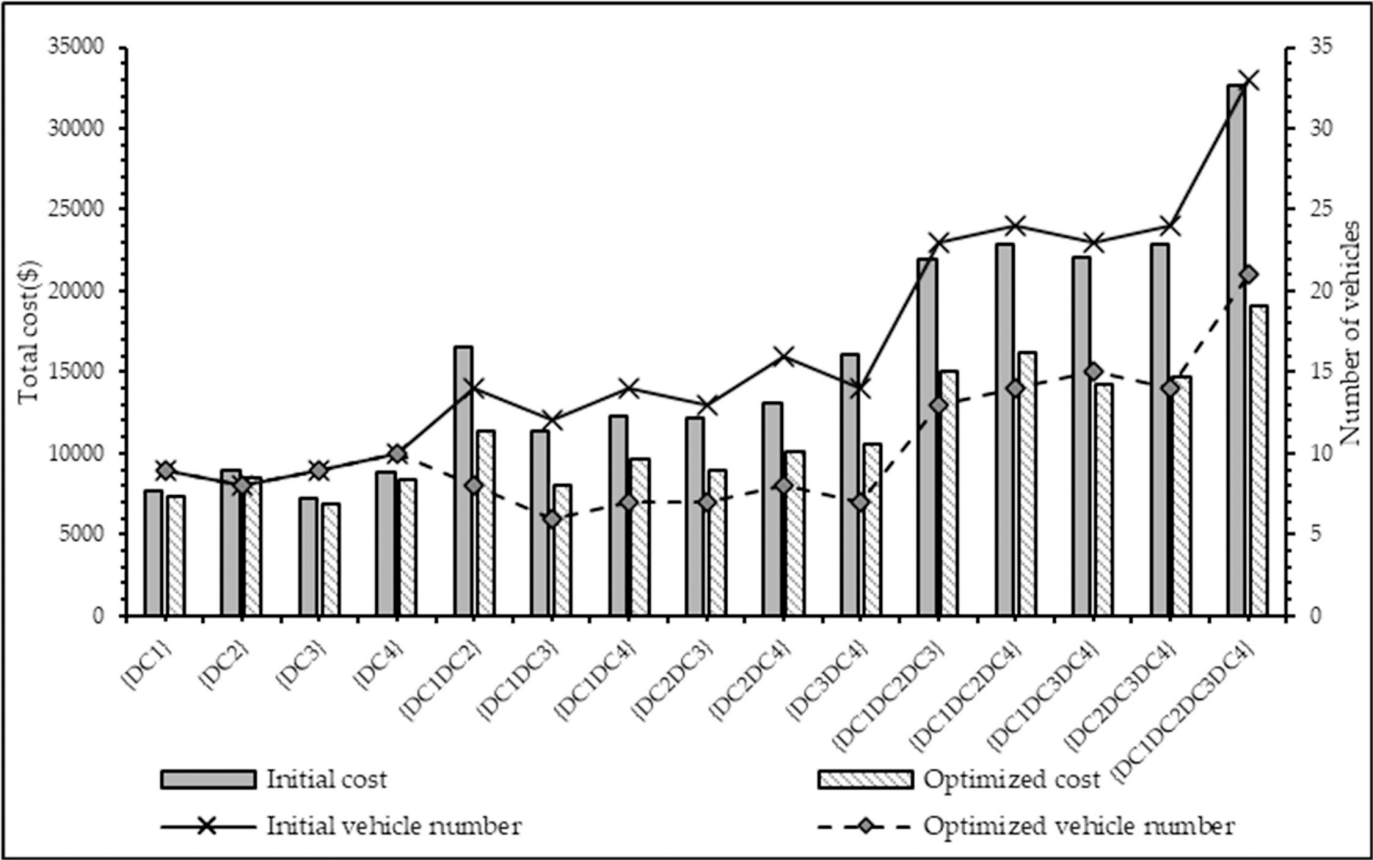
Comparison of initial and optimized costs and number of vehicles.

The optimization results, including cost savings and the changes in initial and optimized costs and number of vehicles, are summarized in Table 13 and illustrated in Fig 5. Table 12 displays that the total costs will be decreased when the participant agrees to join an alliance. For example, the initial costs of alliance {DC1, DC2} is $16551, whereas the optimized cost is $11344, thereby generating a cost savings of $4382. The total cost savings are also affected by the number of alliance members. The cost savings of alliance {DC1, DC2} is $5207, whereas the cost savings of {DC1, DC2, DC3} are $6888. The addition of DC3 saves considerable costs. The number of vehicles is shown in Fig 5, which indicates that the number of vehicles used in the initial alliance are more than the number in the optimized alliance.

**Table 13:** Comparison between initial and optimized networks within one working period.

*A*	Initial		Optimized		*V(A)*
	Cost ($)	Number of vehicles	Cost($)	Number of vehicles	
{DC1}	7659	9	7276	9	383
{DC2}	8892	8	8447	8	445
{DC3}	7223	9	6862	9	361
{DC4}	8825	10	8383	10	442
{DC1DC2}	16551	14	11344	8	5207
{DC1DC3}	11317	12	8026	6	3291
{DC1DC4}	12236	14	9636	7	2600
{DC2DC3}	12165	13	8988	7	3177
{DC2DC4}	13084	16	10138	8	2946
{DC3DC4}	16047	14	10608	7	5439
{DC1DC2DC3}	21905	23	15017	13	6888
{DC1DC2DC4}	22825	24	16196	14	6629
{DC1DC3DC4}	22010	23	14226	15	7784
{DC2DC3DC4}	22858	24	14750	14	8108
{DC1DC2DC3DC4}	32598	33	19089	21	13509

In all circumstances, we observe effective cost reduction in Table 13 and Fig 5. Therefore, DCs should cooperate and share their resources to minimize expenses. Fig 5 indicates that the largest cost and vehicle gaps are generated when forming the grand alliance. Although the collaboration with any other DC is cost effective, the participants intend to increase their profit by considering global sharing and forming a grand alliance. In addition, the logistics provider as the coordinator would not be allocated any profits, and so the synergy requirement parameter *σ* = 0. The cost savings are equally allocated among DC1, DC2, DC3, and DC4 using MCRS. Table 14 shows the allocation results.

**Table 14:** Profit distribution of DCs for global sharing.

*A*	*V(A)*	*X*(*A*,*v*)
{DC1}	383	(383, 0, 0, 0)
{DC2}	445	(0, 445, 0, 0)
{DC3}	361	(0, 0, 361, 0)
{DC4}	442	(0, 0, 0, 442)
{DC1, DC2}	5207	(2573, 2634, 0, 0)
{DC1, DC3}	3291	(1661, 0, 1630, 0)
{DC1, DC4}	2600	(1684, 0, 0, 916)
{DC2, DC3}	3177	(0, 1625, 1552, 0)
{DC2, DC4}	2946	(0, 1487, 0, 1460)
{DC3, DC4}	5439	(0, 0, 2679, 2760)
{DC1, DC2, DC3}	6890	(2592, 2653, 1644, 0)
{DC1, DC2, DC4}	6630	(2512, 2573, 0, 1546)
{DC1, DC3, DC4}	7784	(1979, 0, 3159, 2645)
{DC2, DC3, DC4}	8108	(0, 1967, 3041, 3100)
{DC1, DC2, DC3, DC4}	13509	(2987, 3199, 3703, 3620)

Table 14 shows that different DCs gain different profits in different alliance scenarios. For example, the profit of DC1 for cooperating with DC2 is $2170, whereas its profit for cooperating with DC3 is $1661. In practice, the DC attached to any alliance is subjected to profit gaining from the entire network. Most economically driven companies participate in the alliance to maximize profit. The order in which participants join the alliance will affect the results of the profit allocation [66]. SMP is a method to find the proper alliance sequence for maximizing benefits [48]. In accordance with SMP [24], the feasible alliance sequences are shown in Table 15.

**Table 15:** Feasible alliance based on global sharing in CMCLDN-RS.

*ϕ*_1_(*DC*1,*DC*2,*DC*3,*DC*4)					*ϕ*_2_(*DC*1,*DC3*,*DC2*,*DC*4)				
Participant *i*	DC1	DC2	DC3	DC4	Participant *i*	DC1	DC3	DC2	DC4
*η*(*i*,*ϕ*,1)	5.0%				*η*(*i*,*ϕ*,1)	5.0%			
*η*(*i*,*ϕ*,2)	33.6%	29.6%			*η*(*i*,*ϕ*,2)	21.7%	22.6%		
*η*(*i*,*ϕ*,3)	34.6%	29.8%	22.8%		*η*(*i*,*ϕ*,3)	34.6%	22.8%	29.8%	
*η*(*i*,*ϕ*,4)	39.0%	36.0%	51.3%	41.0%	*η*(*i*,*ϕ*,4)	39.0%	51.3%	36.0%	41.0%
*ϕ*_3_(*DC*1,*DC4*,*DC2*,*DC*3)					*ϕ*_4_(*DC2*,*DC3*,*DC1*,*DC*4)				
Participant *i*	DC1	DC4	DC2	DC3	Participant *i*	DC2	DC3	DC1	DC4
*η*(*i*,*ϕ*,1)	5.0%				*η*(*i*,*ϕ*,1)	5.0%			
*η*(*i*,*ϕ*,2)	22.0%	18.5%			*η*(*i*,*ϕ*,2)	18.3%	21.5%		
*η*(*i*,*ϕ*,3)	33.6%	17.5%	28.9%		*η*(*i*,*ϕ*,3)	29.8%	22.8%	34.6%	
*η*(*i*,*ϕ*,4)	39.0%	41.0%	36.0%	51.3%	*η*(*i*,*ϕ*,4)	36.0%	51.3%	39.0%	41.0%
*ϕ*_5_(*DC*2,*DC4*,*DC1*,*DC3*)					*ϕ*_6_(*DC3*,*DC4*,*DC2*,*DC1*)				
Participant *i*	DC2	DC4	DC1	DC3	Participant *i*	DC3	DC4	DC2	DC1
*η*(*i*,*ϕ*,1)	5.0%				*η*(*i*,*ϕ*,1)	5.0%			
*η*(*i*,*ϕ*,2)	16.7%	16.5%			*η*(*i*,*ϕ*,2)	37.1%	31.3%		
*η*(*i*,*ϕ*,3)	28.9%	17.5%	33.6%		*η*(*i*,*ϕ*,3)	42.1%	35.1%	22.1%	
*η*(*i*,*ϕ*,4)	36.0%	41.0%	39.0%	51.3%	*η*(*i*,*ϕ*,4)	51.3%	41.0%	36.0%	39.0%

Comparisons of all the feasible collaboration sequences in Table 15 indicate that the optimal alliance of the collaborative network is *ϕ*_1_ = {DC1,DC2,DC3,DC4}, as shown in Table 16. In greater detail, the optimal collaboration strategy can be described as follows: DC1 joins the alliance first and achieves 5% cost reduction; DC2 follows and yields 29.6% reduction, and DC1 has 33.6% reduction; when DC3 joins, the reduction becomes 34.6%, 29.8%, and 22.8% for DC1, DC2, and DC3, respectively; and as DC4 enters, DC1, DC2, DC3, and DC4 can reduce their costs by 39.0%, 36.0%, 51.3%, and 41.0%, respectively.

**Table 16:** Optimal collaboration sequences based on the SMP principle.

*ϕ*_1_ = {DC1,DC2,DC3,DC4}				
Participant *i*	DC1	DC2	DC3	DC4
*η*(*i*,*ϕ*,1)	5.0%			
*η*(*i*,*ϕ*,2)	33.6%	29.6%		
*η*(*i*,*ϕ*,3)	34.6%	29.8%	22.8%	
*η*(*i*,*ϕ*,4)	39.0%	36.0%	51.3%	41.0%

**Fig 6:**
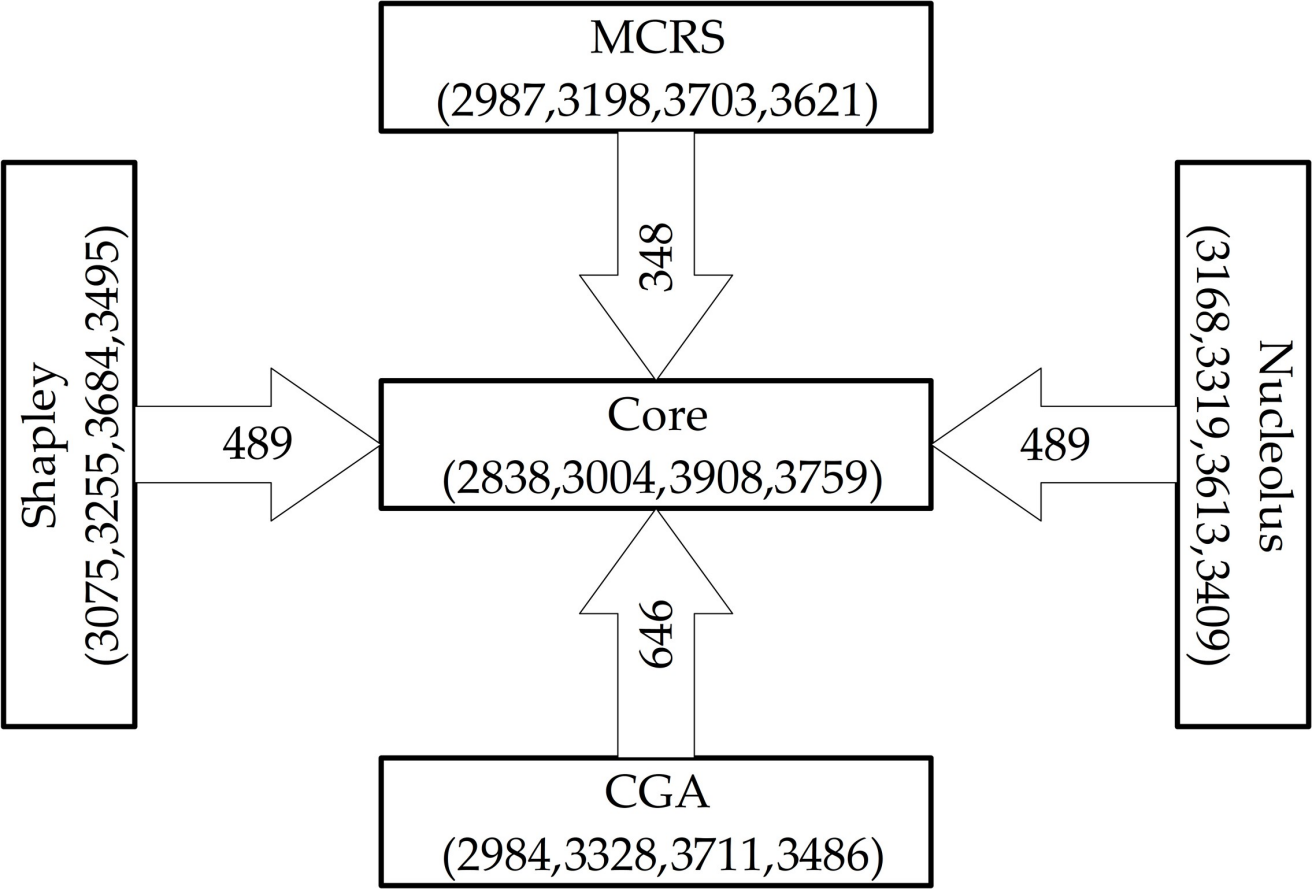
Profit allocation by using MCRS, Shapley, nucleolus, and CGA.

To verify the accuracy of our profit distribution, the Shapley value model, nucleolus, and cost gap allocation (CGA) are applied to find distribution schemes corresponding to the grand alliance and are then compared with MCRS. Considering previous research [67], the core center shown in Fig 6 is also obtained by compressing the polyhedron and narrowing the core into a single point.

Comparisons of the distance between each profit allocation method and the core center prove that CGA is the farthest from the core center and MCRS is the closest. Therefore, the best profit allocation scheme among DCs is $2584, $2777, $2565, and $2448, and MCRS is selected to be the most appropriate allocation strategy in this case study.

## Analysis and discussion

Above we discussed two methods of collaboration to solve the optimization problem of CMCLDN-RS. In this subsection, some analyses and comparisons are made to help find a suitable collaborative mechanism. Table 17 shows the difference between three types of delivery networks: (1) non-collaborative delivery network without vehicle sharing (Case A), (2) collaborative delivery network considering vehicle sharing only within each DC (Case B), and (3) A collaborative delivery network in consideration of vehicle sharing within each DC and among DCs (Case C).

**Table 17:** Comparison of cases A, B, and C.

Scenario	Cost of Case A ($)	Cost of Case B ($)	Cost of Case C ($)	Number of vehicles in Case A	Number of vehicles in Case B	Number of vehicles in Case C
Before optimization	32598	32598	32598	33	33	33
After optimization	29338	22225	19089	31	27	21
Gap	3260	10373	13509	2	6	12

**Fig 7:**
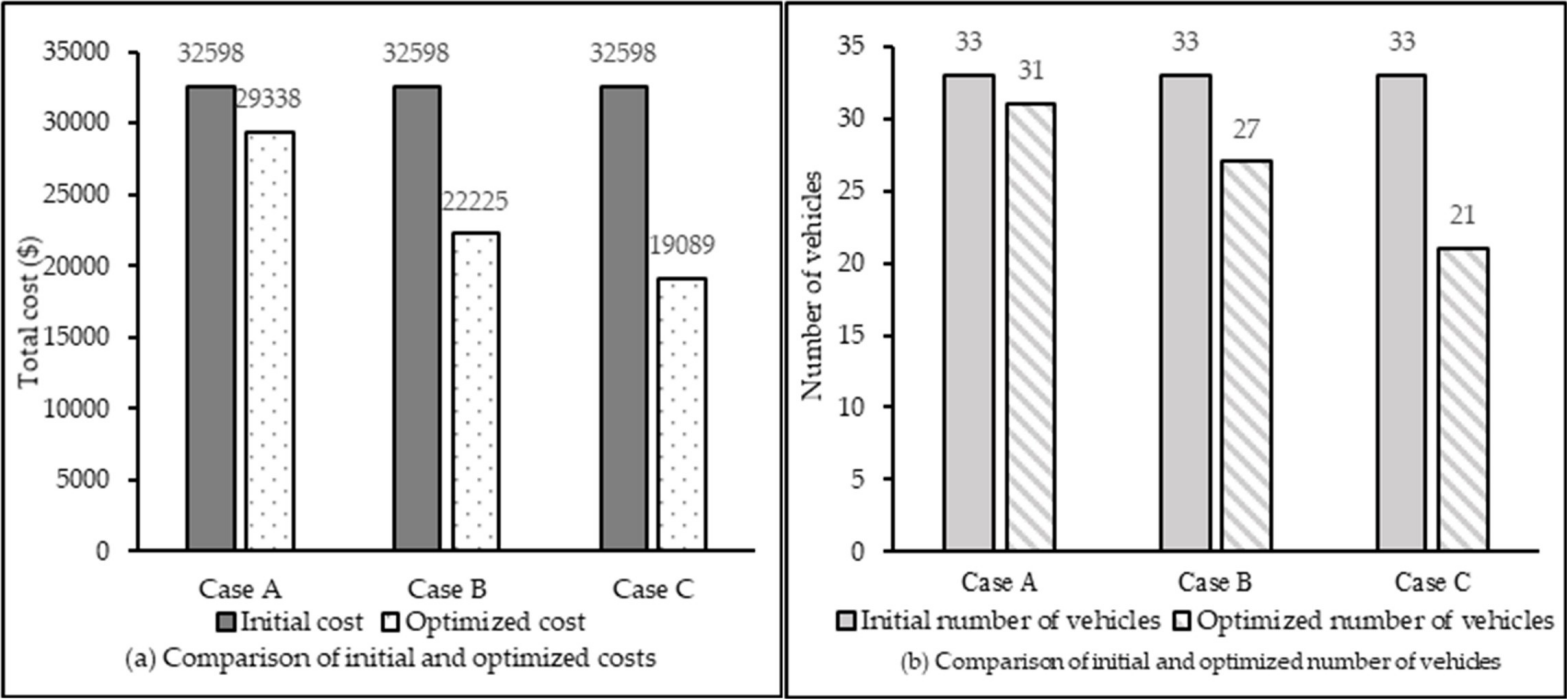
Illustration of cost and number of vehicles in cases A, B, and C.

The two proposed collaboration methods can save the cost and number of vehicles in the optimized logistics network. As shown in Table 17 and Fig 7, significant reductions in total cost and number of vehicles can be achieved. The total logistics operational cost savings are $3260 in Case A and $10373 in Case B. After the CMCLDN-RS optimization, the cost savings increase to $13509 in Case C. The greatest reduction in total number of vehicles is in Case C, from 33 to 21. Therefore, the resource sharing schemes implemented in Case C are much more useful and efficient for optimizing CMCLDN-RS. Table 18 shows the profits of each DC after optimization.

**Table 18:** Profits for each DC in cases A, B, and C.

Participant *i*	Profits in Case A	Profits in Case B	Profits in Case C
DC1	383	2584	2987
DC2	445	2777	3199
DC3	361	2565	3703
DC4	442	2448	3621
Total	1631	10373	13509

**Fig 8:**
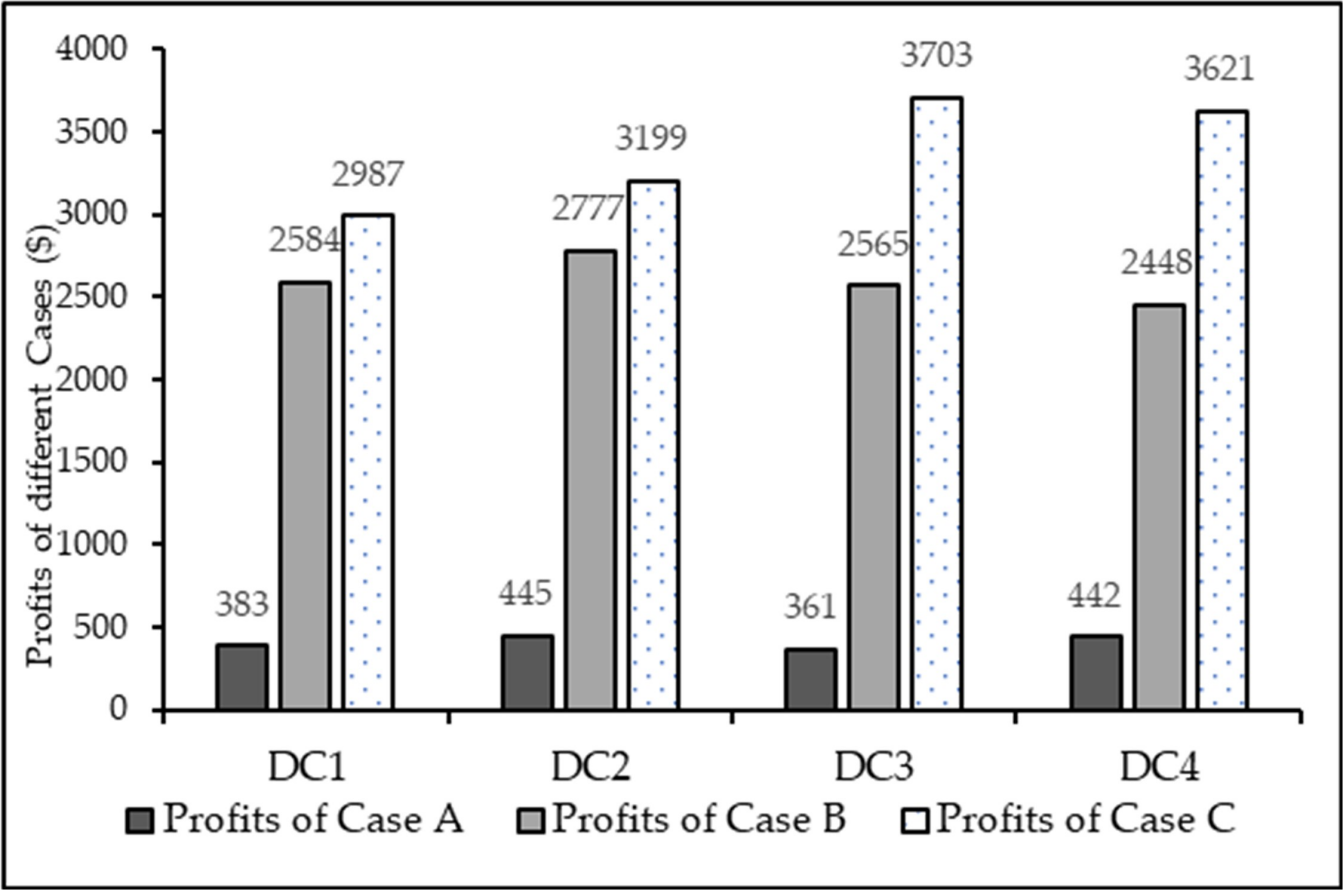
Comparison of profits for each DC in cases A, B, and C.

Table 18 and Fig 8 indicate an obvious profit gap between non-collaborative and collaborative networks for each DC but a relatively small profit gap between Cases B and C. This study considers that the customer demands for special commodities are less than the demands for ordinary commodities. Thus, the cost savings of the component based on vehicle sharing among DCs are relatively small, resulting in low profits for DCs in this part. As shown in Fig 8, the profits for DC1 are $2584 in Case B and $2987 in Case C. The profit gap is $403. However, from a long-term perspective, with the increase of collaboration and the increasing demand for special commodities, the scenario of global vehicle sharing including two types of vehicle sharing schemes is effective.

### Management insights

The CMCLDN-RS optimization provides a reference for the planning of a multicenter logistics network and the improvement of the modern logistics system, which incorporates collaboration among multiple centers and types of resource sharing schemes. Governments and logistics companies can collaborate to integrate logistics resources to improve the efficiency of logistics networks. The managerial insights are provided as follows.

1. Considering different resource sharing schemes in a collaborative multicenter logistics network has far-reaching impacts. The proposed models and intelligent algorithms reduce the total operational cost by $13509 and the number of vehicles by 12 for CMCLDN-RS, which provides a reference for solving the problem of existing different resource sharing schemes in the logistics network. Different resource sharing schemes are suitable for diverse scenarios. A suitable sharing mode can utilize limited logistics resources, improve the utilization rate of resource sharing, and enhance the stability of a cooperative alliance. Therefore, different resource sharing schemes for the rational use of social resources and the development of urban logistics should be considered when designing a logistics network.
2. In the delivery process, a reasonable and effective collaborative mechanism can effectively improve the operational efficiency of the logistics network and reduce the network complexity. The collaborative mechanism can rationally allocate limited resources and achieve resource coordination in the distribution process. Unreasonable transportation phenomena in the non-collaborative logistics network can be effectively avoided by rational division of time periods and rational utilization of resources.

## Conclusions

This study evaluates the optimization of a collaborative network on the basis of resource sharing and aims to find the most suitable collaborative delivery mechanism by exploring the diverse collaboration methods among multiple centers. This collaborative mechanism can not only effectively reduce the total operational costs of the collaborative network but also reduce the number of vehicles used to serve customers. The most suitable collaborative mechanism can be obtained by comparing three types of collaborative methods in different logistics networks, including non-collaborative networks, internally shared collaborative networks, and globally shared collaborative networks. A mixed-integer linear programing model is established to minimize the total operational costs. An improved MOPSO algorithm is proposed to solve the optimization problem. A fair profit strategy is presented to allocate the profits to alliance participants.

To verify the applicability of the proposed CMCLDN-RS in real life, a case study is conducted in the city of Chengdu, China. The total operational costs and number of vehicles before and after CMCLDN-RS optimization reduce significantly as a result. After the implementation of a collaborative mechanism based on resource sharing, the total operational costs are effectively reduced from $32598 to $19089, indicating savings of $13509; in addition, the number of vehicles changed from 33 to 21, implying a reduction of 12 vehicles. Comparison among AGPSO, NSGA-II, and GA-TS indicates that AGPSO performs best among the three in terms of solution quality. Among the four profit allocation methods of MCRS, Shapley, nucleolus, and CGA, MCRS is proven to be the most appropriate profit allocation method for fairly allocating the cost savings to participants.

This study measures the effects of different collaboration modes on the delivery network, an area that needs additional research. Future work can be conducted in the following four directions by considering: (1) different collaboration methods among pickup centers and DCs on the basis of resource sharing; (2) the simultaneous pickup and delivery problem in vehicle sharing; (3) the effect of vehicle space on delivery network optimization; and (4) the dynamic and uncertain customer demands during each vehicle delivery trip in a multi-echelon collaborative network.

## Supporting information

S1 Dataset The dataset includes the longitude and latitude coordinates and service time windows of all customers and four DCs in case study. (XLSX)
